# Letter from Elgin

**Published:** 1882-07

**Authors:** H. Rozencrans

**Affiliations:** Elgin, Ill.


### ARTICLE X.

ELGIN, ILL., FEB. 10, 1882.

EDITORS MEDICAL JOURNAL AND EXAMINER:

In as brief a manner as possible, I will relate to you two railroad accidents occurring under my observation and treatment while in Indianola, Texas. It must be observed that in my surgical operations while there I had to depend on but little help, as help was not to be had. The first of these two cases was a Mr. J., twenty-one years of age, who, while in the act of coupling the cars together, met with the accident of having his right arm badly broken and lacerated, also one of his feet was injured, requiring the amputation of one toe. Three days after the accident his arm began rapidly to mortify, requiring amputation at the shoulder without further delay. I only had the assistance of a timid doctor, who begged me not to undertake the job, the patient begging me to save his life if possible. I chloroformed him and amputated at the shoulder joint, and saved his life. This accident occurred in December, 1878.

In the winter of 1879, on Christmas day, J. R., roadmaster, thirty years of age, while on his way to Victoria with the train, stopping at the first station, twelve miles out from Indianola, while in the act of stepping on the train as it was about to start, made a misstep, and one of the wheels passed over his foot, mashing and lacerating it badly. As the train was on its way to Victoria, he was taken on there, and I was at once telegraphed to come up, distance fifty miles; a special car took me up. I found his injury such that I was sure an amputation was necessary, but the matter was delayed two days, and I then fixed up a swing bed on one of the cars and took him to Indianola, and the next day telegraphed to Dr. Sutherland, of Victoria, to come down and assist me in amputating his leg. Mortification had commenced, spreading over the foot, and was extending up the leg. The patient was made ready and Dr. S. administered the chloroform, and I at once amputated just below the knee. I noticed the cut muscles were quite dark-colored, and I feared a second amputation above the knee would be necessary, but I succeeded in healing up the stump, making a good one, but not by the first intention. I afterward took measurement of the stump, and sent to New York and succeeded in getting him a good fit of an artificial limb. H. ROZENCRANS, M.D.

